# Effect of Expressive Writing Intervention on Health Outcomes in Breast Cancer Patients: A Systematic Review and Meta-Analysis of Randomized Controlled Trials

**DOI:** 10.1371/journal.pone.0131802

**Published:** 2015-07-07

**Authors:** Chunlan Zhou, Yanni Wu, Shengli An, Xiaojin Li

**Affiliations:** 1 Nanfang Hospital, Southern Medical University, Guangzhou, Guangdong, PR China; 2 Department of Bio-Statistics, Guangdong Provincial Key Laboratory of Tropical Disease Research, School of Public Health and Tropical Medicine, Southern Medical University, Guangzhou, Guangdong, PR China; University of St Andrews, UNITED KINGDOM

## Abstract

**Background:**

Numerous randomized controlled trials (RCTs) have arrived at conflicting conclusions on expressive writing (EW) as an intervention for breast cancer (BC) patients, but there has been no meta-analysis of these studies to assess the effectiveness of EW in BC population.

**Methods:**

PubMed, Web of Science, The Cochrane Library, EMBASE, and CINAHL and the www.clinicaltrial.gov database on ongoing clinical trials were searched to identify all the RCTs investigating efficacy of EW on the physical and psychological health in BC patients. The risk of bias of the original studies was assessed using the Cochrane Collaboration’s tool. Our primary outcomes for physical and psychological health were respectively negative somatic symptoms and negative mood which were stratified by emotional, benefit-finding and multiple prompts in sub-group analyses. The data were analyzed using Review Manager 5.2 and Stata version 12.0 statistical software.

**Results:**

Of the 5,232 titles screened, we identified 11 RCTs with a total of 1,178 participants. The pooled results showed a significant effect of EW using either an emotional prompt or a benefit-finding prompt on reducing negative somatic symptoms in BC patients in the ≤3-month follow-up group [Mean Difference (MD), -13.03, 95% CI, -19.23 to -6.83, P<0.0001; MD, -9.18, 95% CI, -15.57 to -2.79, P = 0.005]. There was no significant effect of EW on physical health in the >3-month follow-up group. There were no significant differences regarding psychological health indexes between EW intervention and control groups at any of the follow-up time-points (P>0.05).

**Conclusion:**

This systematic review and meta-analysis reveals that EW intervention may have a significantly positive impact on the physical health but not the psychological health in BC patients, but this benefit may not last long. However, further high-quality studies with more homogeneity are needed to confirm the current findings.

## Introduction

Emotional expression, as a psychological or medical intervention, has been studied for many years, demonstrating favorable impacts on physical and mental health [1–7]. Expressive writing (EW) as a form of emotional expression was first implemented in college students in 1986 by Pennebaker and Beall, who instructed respondents to write about their deepest emotions and thoughts regarding traumatic/upsetting experiences for approximately 20 minutes over four consecutive days [8]. Afterwards, work on EW as a potential intervention for physical and psychosocial adjustment was extended to clinical and medical populations, including non-patients [9–12] as well as patients with rheumatoid arthritis [13,14], asthma [15], HIV [16], cardiovascular disease [17] or renal cell carcinoma [18]. Since Walker explored the feasibility of using EW in a breast cancer (BC) cohort [19], there have been numerous randomized controlled trials (RCTs) testing effectiveness of EW in BC patients [20–29]. These studies have been performed for reasons like: BC remains the second most frequently diagnosed type of cancer in women [30], many BC patients report feeling emotionally inhibited which has been linked with worse psychological functioning [1,31,32], and physical problems are still highly prevalent in BC population [33–38].

Although EW was generally considered beneficial, some researchers questioned its utility in light of failures to replicate the original findings [15,39]. Some studies reached a negative conclusion on the benefits of EW [40–44] while others demonstrated positive effects of EW on the physical and psychological health in various populations, mostly patients [13,16,45–49]. Systematic reviews of the studies on the efficacy of EW in healthy and unhealthy populations also led to various conclusions [50–55]. Diversified concerns, inclusion of different populations, measurement of different variables and different methodology may have been significant reasons for their inconsistent findings. Harris and Mogk concluded that EW had little effect on the subjects tested [53,54], Frisina and Frattaroli found EW was effective [50,52], but Boinon and Merz could not make a definite conclusion about EW in cancer patients [51,55]. Similarly, studies on EW in BC patients also arrived at conflicting conclusions. Some studies failed to confirm the benefit of EW [19,22,23,26], but others found positive effects of EW on the physical or psychological health of this particular population [20,21,24,25,27–29].

Our concern focuses on the benefits of EW on the physical and psychological health of BC population. Firstly, EW is a low-cost, convenient and self-administered intervention that can be routinely used in clinic if its therapeutic benefits can be confirmed. Secondly, BC victims are overwhelmingly females who have to brave particular physical and psychological challenges that may impact their therapeutic outcomes after diagnosis and treatment of BC [56–63]. There is a great demand to develop all kinds of interventions which may help them cope with their specific physical and psychological challenges on their way to combat BC. Moreover, yearly increase of more than 1.3 million new cases has made BC the most frequently diagnosed cancer in women worldwide [64]. However, numerous studies on EW in BC patients have provided inconsistent findings and there has been no meta-analysis of these studies so far. According to the literature published, although Boinon and Merz both conducted a systematic review [51,55] of the effectiveness of EW in cancer patients, they did not focus their concern specifically on BC population and did not perform meta-analysis. Therefore, it is necessary to perform a meta-analysis to review all the data from all the high-quality studies available on this topic to make convincing up-to-date conclusions about EW in BC population. This study aimed to test the hypothesis that EW might be a promising clinical intervention to improve the physical and psychological health in BC patients by determining whether EW was beneficial for BC patients, what were the benefits, and how effective was EW in relieving physical and psychological symptoms.

## Methods

### Search methods

We searched the following databases to identify relevant studies for this meta-analysis and adapted different search strategies according to the query requirements of the individual databases. We limited our search by the time after the year 1986 when the first EW study using Pennebaker’s prompt was published. We did not restrict our search by language. The following databases were queried: PubMed (from 1986 to June 2014), Web of Science (from 1986 to June 2014), The Cochrane Library (from 1986 to June 2014), EMBASE (from 1986 to June 2014), and CINAHL (from 1986 to June 2014) (S1 Appendix). We searched the database of ongoing trials, www.clinicaltrial.gov. We also screened the references of included studies to identify additional articles. We did not handsearch journals or conference proceedings, due to limited time and resources.

### Inclusion criteria

To ensure homogeneity across studies, we included studies that met the following criteria: (1) a randomized controlled trial (RCT) with an experimental design that included an EW group (expressive writing for at least a single 20-minute session using the Pennebaker and Beall paradigm [8]) and a control group for comparison; (2) women participants with a BC diagnosis, irrespective of their age, BC stage, treatment modality or treatment setting (including inpatient, outpatient and primary care); (3) outcome measures that assessed factors relative to the physical and psychological health of BC patients. We excluded review articles or studies the complete data of which were unavailable.

### Assessment of methodological quality

The quality of included studies was assessed using the Cochrane Collaboration’s risk of bias tool. Each study was assessed for random sequence generation (selection bias), allocation concealment (selection bias), blinding of participants and personnel (performance bias), blinding of outcome assessment (detection bias), incomplete outcome data (attrition bias), selective reporting (reporting bias), and any other potential sources of bias. Each factor would be rated as “low risk” of bias (e.g., random sequence generation was computer generated), “high risk” of bias (e.g., outcome assessment was not blinded) or “unclear risk” of bias (e.g., did not provide specific information as to whether allocation concealment was used). Disagreements would be resolved by consensus.

### Data extraction

Two authors (YNW and XJL) independently extracted the data from each trial using a standardized data extraction form that included general information (author, title, source, contact address, and year of publication), the trial characteristics (randomization method, blinding, duration of intervention period, length of follow-up, and method for handling missing data), the patient characteristics (sample size, stage of disease, race, age, level of education, average time since diagnosis, and inclusion criteria), the intervention (detailed description of the controlled intervention, mode, and duration) and outcomes (outcome measures and scoring range). When data were missing, one author (YNW) contacted the authors to request additional information. If further information could not be obtained, we coded the variables in question as “NR”.

### Data analysis

We used Review Manager 5.2 (Cochrane Collaboration, Oxford, UK) and Stata version 12.0 (Stata Corp, College Station, Texas, USA) for data analysis. Two investigators (YNW and SLA) were involved in the statistical analysis. Measurement of outcomes was considered in terms of original data at each follow-up time point (baseline scores not included). The mean difference (MD) and 95% CI were calculated based on fixed-effect model for continuous variables. The z-test was used to obtain the combined P-values of the included studies with a significance level of P = 0.05. The statistical significance of heterogeneity among studies was assessed by calculating the chi-square test (a P-value of 0.10 was regarded as statistically significant). The I^2^ was used to quantify the effects of heterogeneity. If statistical heterogeneity (P value ≤0.10 and I^2^≥50%) was identified, random effects meta-analysis was conducted before the causes of heterogeneity was further investigated by subgroup analysis; if not, a fixed-effects model was used [65]. Egger’s test and funnel plot were conducted to investigate the potential publication bias influencing the analysis. To determine whether significant differences would exist between specific variables regarding the effectiveness of EW, subgroup analyses were carried out by sorting the same specific variables reported in the RCTs.

## Results

### Description of the studies

#### Search results

**Fig 1:**
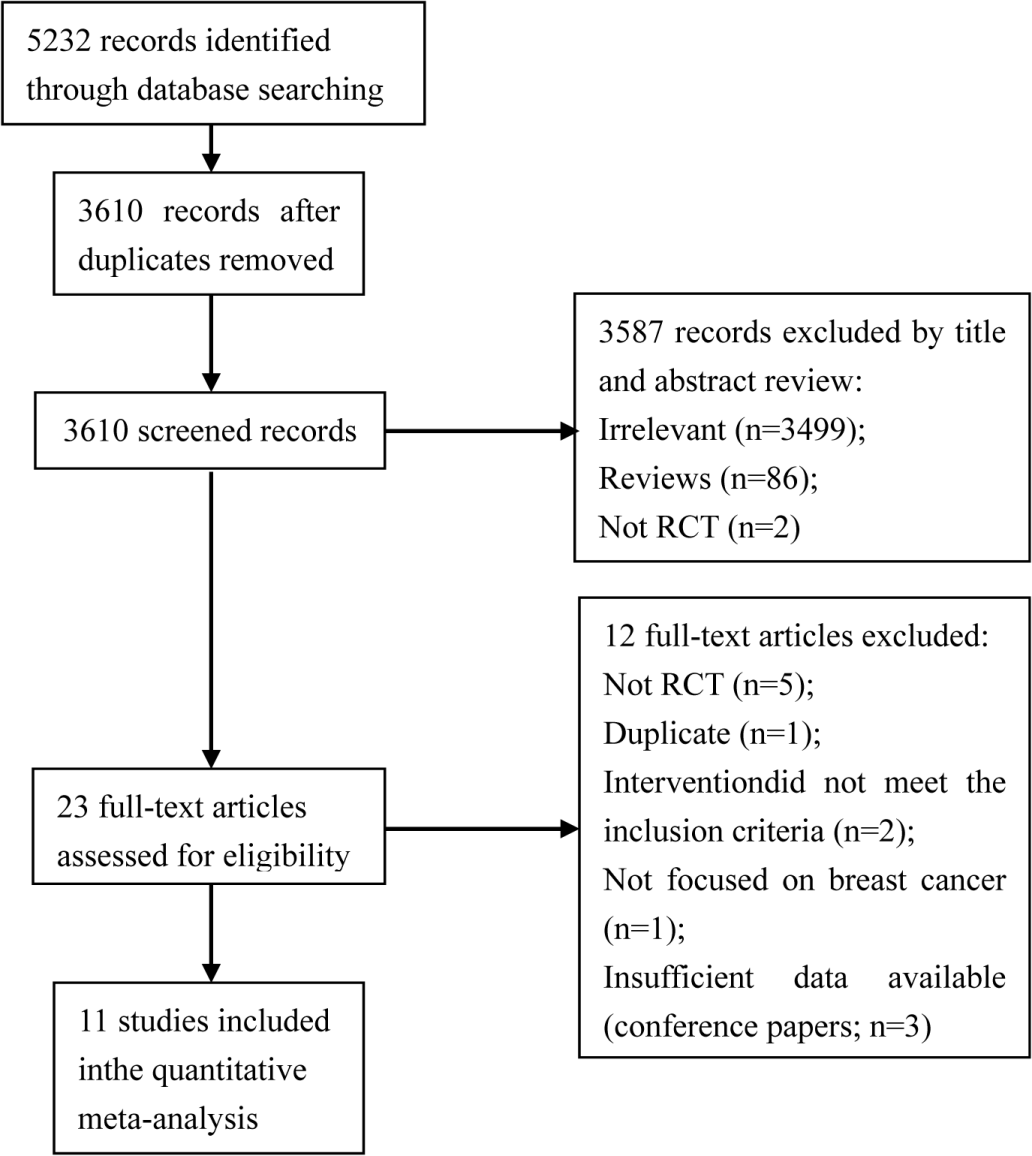
Study flow diagram.

We conducted the electronic searches in June 2014. A total of 5232 titles and abstracts were screened, and 1622 duplicates were identified. Of the 3610 screened titles and abstracts, 3587 were excluded. After we read the remaining 23 full-text articles, 12 full-text articles were excluded [66–77] and 11 studies included. No additional studies were identified by searching the reference lists. There were no ongoing studies that we were aware of. The study flow diagram is illustrated in Fig 1.

#### Included studies

Eleven studies examining the effectiveness of EW as an intervention on the health outcomes in BC patients were included in this meta-analysis [19–29]. Descriptions of the studies and samples are presented in Table 1.

**Table 1:** Characteristics of the included studies

Reference (year)	Sample	Breast cancer stages	Race	Mean age (years)	College educated	Average time since diagnosis	Task	Follow-up	Outcomes
Craft et al. (2013)	97	0–3	92.8% Caucasian	56±10.5	NR	13 months	*EW*: EMO (*n* = 26), EMO^+^ (*n* = 19); *Control*: NW_c_ (*n* = 22), USUAL (*n* = 30); Four sessions, consecutive days	1 and 6 months	Aspects of psychological health measured using: 1. Functional Assessment of Cancer Therapy-Breast Cancer Version (FACT-B)
De Moor et al. (2008)	49	2–3	63% Caucasian	53.5±10.4	78.5%	NR (had finished neoadjuvant chemotherapy)	*EW*: EMO (*n* = 24); *Control*: NW_b_ (*n* = 25); Four sessions over 7 days, 3 weeks prior to surgery	3 days pre-surgery and 2 weeks post-surgery	Aspects of physical health measured using: 1. Brief Pain Inventory (BPI), 2. Pittsburgh Sleep Quality Index (PSQI); Aspects of psychological health measured using: 1. Brief Symptom Inventory 18 (BSI-18), 2. Perceived Stress Scale (PSS)
Gellaitry et al. (2010)	80	1–2	NR	57.9±9.9	NR	NR (had received radiotherapy treatment)	*EW*: MULTIPLE (*n* = 38); *Control*: USUAL (*n* = 42); Four sessions, consecutive days (different prompt each day)	1, 3, and 6 months	Aspects of physical health measured using: 1. Healthcare utilization; Aspects of psychological health measured using: 1. Profile of Mood States (POMS), 2. Functional Assessment of Cancer Therapy-Breast Cancer Version (FACT-B)
Henry et al. (2010)	80	1–4	NR	58.9±NR	NR	18 months	*EW*: BEN (*n* = 40); *Control*: USUAL (*n* = 40); One session	3 and 9 months	Aspects of physical health measured using: 1. Physical health measure (included 18 physical symptoms); Aspects of psychological health measured using: 1. Center for Epidemiologic Studies-Depression Scale (CES-D), 2. Profile of Mood States (POMS)
Jensen-Johansen et al. (2012)	507	1–2	NR	53.6±9.1	40%	NR (days since surgery 151±55)	*EW*: EMO^+^(*n* = 253); *Control*: NW(*n* = 254); Three sessions over 3 weeks	3 and 9 months	Aspects of psychological health measured using: 1. Impact of Events Scale (IES), 2. Beck Depression Inventory-Short Form (BDI-SF), 3. Profile of Mood States (POMS), 4. Passive Positive Mood Scale(PPMS)
Low et al. (2010)	62	4	87% Caucasian	53.8±10.3	74%	7.9 years	*EW*: EMO (*n* = 31); *Control*: NW_c_ (*n* = 31); Four sessions over 3 weeks	3 months	Aspects of physical health measured using: 1. Negative somatic symptoms scale (developed by Pennebaker, includes 9 somatic symptoms), 2. Pittsburgh Sleep Quality Index (PSQI); Aspects of psychological health measured using: 1. Center for Epidemiologic Studies-Depression Scale (CES-D), 2. Impact of Events Scale (IES)
Low et al. (2006)	60	1–2	NR	NR	NR	NR (had completed primary medical treatments)	*EW*: EMO (*n* = 21), BEN (*n* = 21); *Control*: NW_c_ (*n* = 18); Four sessions over 3 weeks	3 months	Aspects of physical health measured using: 1. Negative somatic symptoms scale (developed by Pennebaker, includes 9 somatic symptoms), 2. Medical appointments for cancer-related morbidities; Aspects of psychological health measured using: 1. Profile of Mood States (POMS)
Mosher et al. (2012)	86	4	81% Caucasian	57.9±12.1	87%	Average time since diagnosis of stage 4 breast cancer: 4 years	*EW*: EMO (*n* = 44); *Control*: NW (*n* = 42); Four sessions over 4–7 weeks	8 weeks	Aspects of physical health measured using: 1. The Functional Assessment of Chronic Illness Therapy Fatigue subscale (FACIT-F), 2. Pittsburgh Sleep Quality Index (PSQI); Aspects of psychological health measured using: 1. The Functional Assessment of Chronic Illness Therapy–Spiritual Well-being scale (FACIT-Sp), 2. Distress Thermometer (DT), 3. Center for Epidemiologic Studies-Depression Scale (CES-D), 4. Anxiety: Hospital Anxiety and Depression Scale (HADS-A)
Park et al. (2012)	58	2–3	NR	48.2±7.5	57%	23 months	*EW*: EMO (*n* = 29); *Control*: USUAL (*n* = 29); Six sessions over 6 weeks	4 weeks	Aspects of physical health measured using: 1. Pennebaker’s Inventory of Limbic Languidness (PILL), 2. M. D. Anderson Symptom Inventory (MDASI); Aspects of psychological health measured using: 1. Hospital Anxiety and Depression Scale (HADS), 2. Cancer-Quality of Life (C-QOL)
Stanton et al. (2002)	60	1–2	93% Caucasian	49.5±12.2	NR (average education level: 15.20±2.48 years)	28.4 weeks	*EW*: EMO (*n* = 21), BEN (*n* = 21); *Control*: NW_c_ (*n* = 18); Four sessions over 3 weeks	1 and 3 months	Aspects of physical health measured using: 1. Negative somatic symptoms scale (developed by Pennebaker, includes 9 somatic symptoms), 2. Medical appointments for cancer-related morbidities; Aspects of psychological health measured using: 1. Functional Assessment of Cancer Therapy (FACT), 2. Profile of Moods State (POMS)
Walker et al. (1999)	39	1–2	95% Caucasian	53.6	79%	NR (were completing RT for stage 1 or 2 breast cancer)	*EW*: 1 session EMO (*n* = 11), 3 sessions EMO (*n* = 14); *Control*: ATT (*n* = 14); One or three sessions over 1–4 days	1, 4–6, 16, and 28 weeks	Aspects of psychological health measured using: 1. Positive and Negative Affect Scale (PANAS), 2. Impact of Events Scale (IES)

EMO, emotional (cancer) prompt; EMO^+^, emotional (any trauma) prompt; BEN, benefit-finding (cancer) prompt; MULTIPLE, emotional, benefit-finding, cognitive-appraisal, and coping prompts; NW, neutral writing (trivial); NW_b_, neutral writing (health behavior); NW_c_, neutral writing (cancer); USUAL, usual care; ATT, non-cancer attention; NR, not reported. The above format is cited in Merz [51].

In all the 11 RCTs, the participants had been randomly divided into an EW group and a control group. The intervention methods in the EW groups included EW with an emotional prompt (cancer or any trauma), a benefit-finding prompt (cancer) or multiple prompts (emotional, benefit-finding, cognitive appraisal and coping strategies), and those in the control groups included neutral writing or no writing (usual care or non-cancer attention).

In total, 1178 BC patients were involved, with 613 in the EW intervention groups and 565 in the control groups. The BC stages ranged from 0 to 4. Six studies reported racial distribution of the participants, with the Caucasian race accounting for more than 60% in three studies and greater than 90% in the other three. Age distribution was provided in 10 studies, with an average age of approximately 50 years. Educational background of the subjects was reported in seven studies, in six of which from 40% to 87% of the subjects had a university or higher degree and in one of which only the average years of education (15.20±2.48) were provided. Five studies reported the time between diagnosis of BC and enrollment of the subjects was between 28.4 weeks and 1.9 years; one study included subjects who were enrolled 4 years after a stage-4 BC diagnosis; one study involved subjects enrolled 151±55 days after operation; the remaining four studies provided no specific time.

The EW intervention in the 11 studies was based on the Pennebaker and Beall [8] paradigm. The number of EW sessions ranged from 1–6 with a minimum of 20 continuous minutes of writing per session. Overall, most of the 11 studies had more than 3 writing sessions. The follow-up time ranged from 1 week to 9 months after the EW intervention, with 3-month follow-up implemented in six studies.

The effect of EW intervention on physical health was evaluated using 11 indexes by eight studies [20–22,24–26,28,29], three of which measured negative somatic symptoms using the negative somatic symptoms scale [78] (Table 1). The effect of EW on psychological health was evaluated using 18 indexes by all the 11 RCTs [19–29], five of which observed negative mood using Profile of Mood States (POMS). Therefore, this meta-analysis decided to take negative somatic symptoms as the primary outcome for physical health and negative mood as the primary outcome for psychological health. The remaining indexes were regarded as the secondary outcomes for either physical health or psychological health, respectively.

#### Risk of bias in the included studies

**Fig 2:**
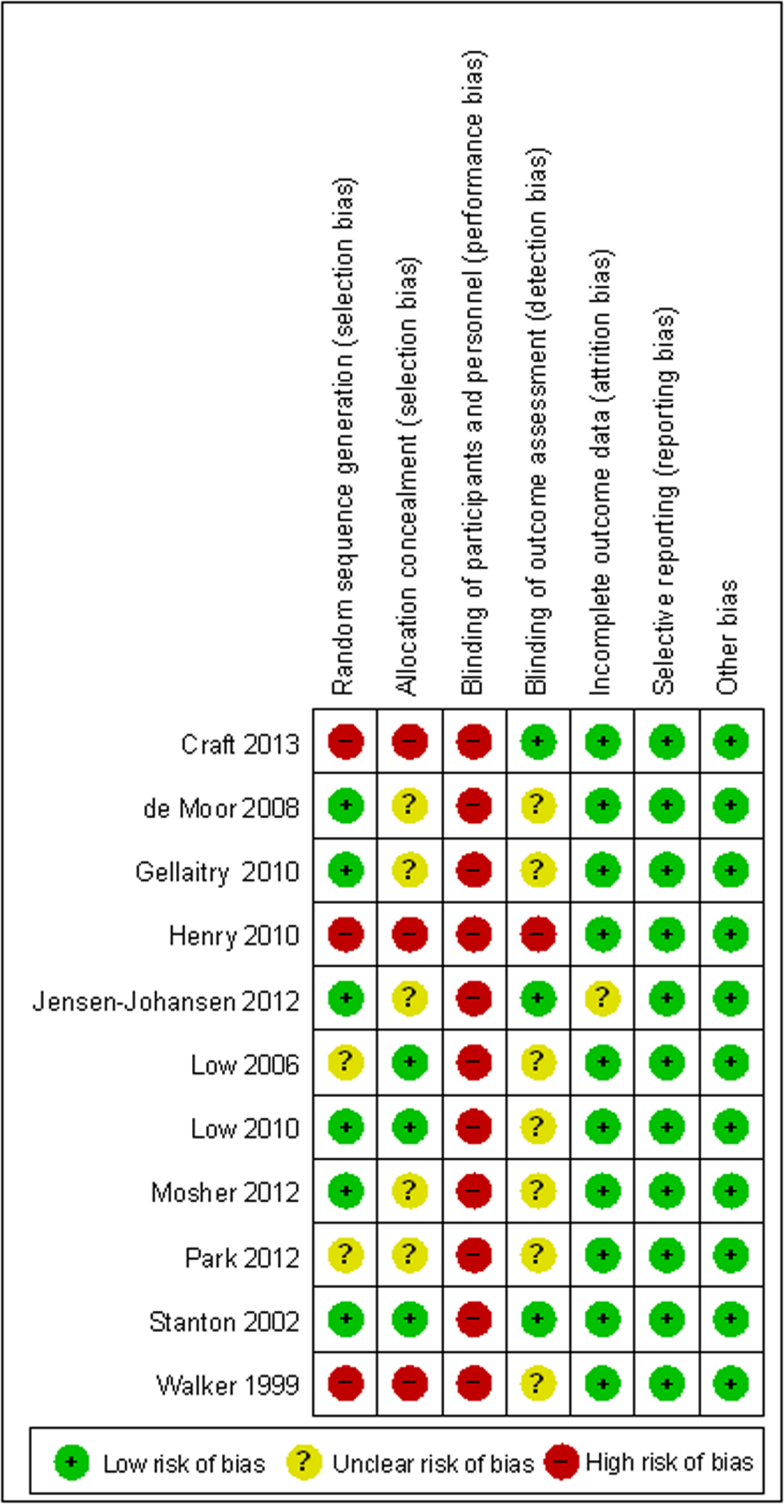
Risk of bias for the included studies.

The risk of bias in the 11 studies was assessed using the Cochrane Collaboration’s tool. The results are summarized in Fig 2.

The selection bias was considered low in six studies because a random serial grouping program was used. Three of them were rated as completely low-risk because they employed a computer-aided serial grouping program [22,25,28]; the random programs in the other three studies were limited in most occasions, thus enhancing the predictability of grouping for the researchers [21,23,26]. Three studies were considered as high-risk of bias because sequential assignment [19,27] or matched controls [24] was utilized. The remaining two studies that did not describe details of their randomization were rated as unclear risk of bias [20,29].

Three RCTs were identified as low selection bias because they used sequentially numbered envelopes to conceal allocation of participants from the researcher or research assistants [22,28,29]. Three RCTs were identified as high selection bias because they did not use condition allocation concealment of participants [19,24,27]. The remaining five studies did not provide specific information as to whether allocation concealment was used.

In the EW intervention, it was difficult to perform a fully blinded study because each researcher and subject had to be informed of the writing requirements and methods. Therefore, the bias risk was high in the 11 studies regarding the blinding of the participants and personnel.

As for blinding of outcome assessment, three studies were considered low-risk because they made efforts to mask the research purposes and writing tasks from research assistants and participants [23,27,28]. One study was considered high-risk because the nurse in charge of the baseline interview and assessment did not use a blinded method for allocation [24]. It was unclear whether a blinded method was implemented in the outcome assessment for the remaining seven studies.

Ten studies that stated the reason why and the time when a subject dropped out exhibited low risk of attrition bias. Notably, Craft [27] used an intent-to-treat analysis to reduce the possibility of data bias. The remaining one study was regarded as unclear risk because no reason was provided for a subject dropping out of the study [23].

All the 11 studies appeared to have reported on all measured outcomes, showing low risk of reporting bias. No other potential sources of bias were noted in the included studies.

### Effects of the interventions

To determine how long the effect of EW intervention would last in BC patients, we divided the pooled outcome measures of physical health and of psychological health respectively into a ≤3-month follow-up group and a >3-month follow-up group. In studies that provided outcome measures of more than one follow-up that met the criteria of grouping, the follow-up data at one time point that satisfied the grouping criteria the most were included while the other data were excluded. Specifically, the data of 3 days pre-surgery [26], 1 week [19] and 1 month [25,28] were excluded from the ≤3-month group while the follow-up results of 16 weeks [19] were excluded from the >3-month follow-up group.

**Fig 3:**
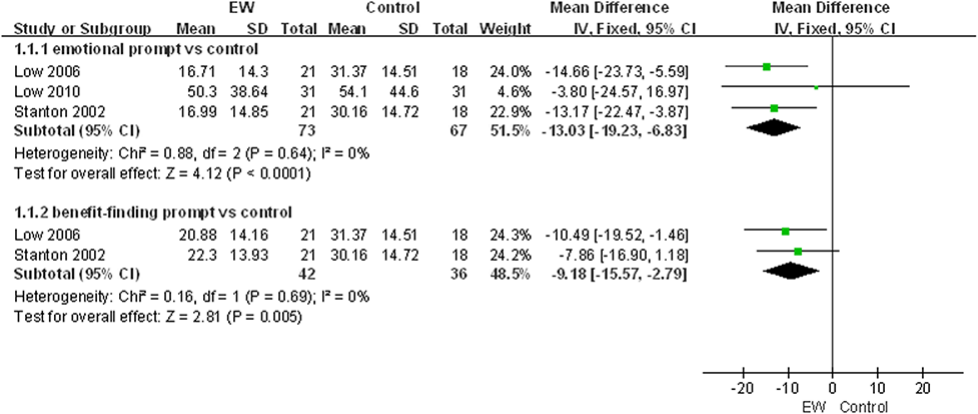
EW intervention and physical health in ≤3-month follow-up group: negative somatic symptoms.

Furthermore, to determine whether different means of EW intervention might have an impact on the effectiveness of EW intervention we stratified the negative somatic symptoms for physical health and negative mood for psychological health, which were primary outcomes for our meta-analysis, by emotional prompt, benefit-finding prompt and multiple prompts. However, since only one study [25] utilized multiple prompts but did not evaluate negative somatic symptoms, actually it was only feasible for us to stratify negative somatic symptoms by emotional prompt and benefit-finding prompt (Fig 3).

#### Expressive writing intervention and physical health

**Fig 4:**
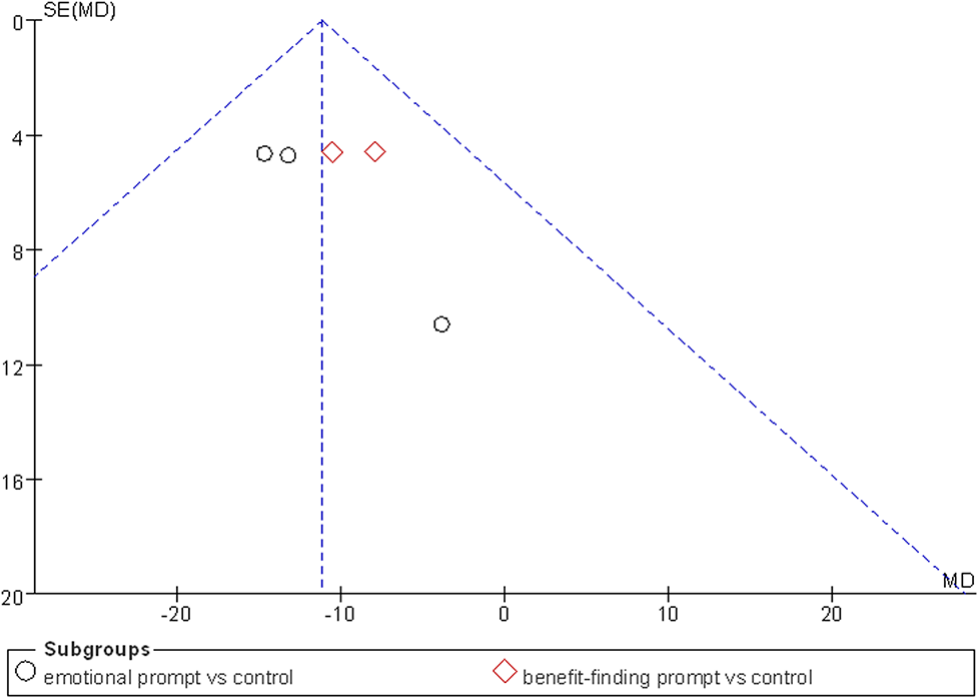
Funnel plot for EW intervention and physical health in ≤3-month follow-up group: negative somatic symptoms.

As shown in Table 2, seven studies in the ≤3-month follow-up group employed a fixed-effect model to analyze the combined effect of EW on the physical health indexes. The results showed a significant effect of EW using either an emotional prompt or a benefit-finding prompt on reducing negative somatic symptoms in BC patients in the ≤3-month follow-up group [Mean Difference (MD), -13.03, 95% CI, -19.23 to -6.83, P<0.0001; MD, -9.18, 95% CI, -15.57 to -2.79, P = 0.005] compared with the control group (Fig 3). A publication bias analysis using a funnel plot was performed on the studies involving these indexes, and the results exhibited a symmetric distribution, indicating a low publication bias (Fig 4). Furthermore, Egger’s test also indicated a low publication bias (P = 0.372).

**Table 2:** EW intervention and physical health in ≤3-month follow-up group.

	Number of		Effect size			Heterogeneity		
Outcome	Comparison	participants	MD	95% Cl	P value	χ^2^	P value	I^2^ (%)
1 Negative somatic symptoms scale, 4 sessions	5			Subtotals only			Subtotals only	
1.1 emotional prompt vs control	3	140	-13.03	-19.23, -6.83	<0.0001	0.88	0.64	0
1.2 benefit-finding prompt vs control	2	78	-9.18	-15.57, -2.79	0.005	0.16	0.69	0
2 Medical appointments for cancer-related morbidities, 4 sessions	4	156	-1.69	-2.30, -1.08	<0.00001	0.99	0.80	0
3 Worst pain: Brief Pain Inventory, 4 sessions	1	49	1.76	0.27, 3.25	0.02	NA	NA	NA
4 Least pain: Brief Pain Inventory, 4 sessions	1	49	0.82	-0.22, 1.86	0.12	NA	NA	NA
5 Average pain: Brief Pain Inventory, 4 sessions	1	49	0.82	-0.34, 1.98	0.16	NA	NA	NA
6 Pain interference: Brief Pain Inventory (BPI), 4 sessions	1	49	1.28	-0.05, 2.61	0.06	NA	NA	NA
7 Pittsburgh Sleep Quality Index (PSQI), 4 sessions	3	199	0.65	-0.15, 1.46	0.11	0.42	0.81	0
8 The Functional Assessment of Chronic Illness Therapy Fatigue subscale (FACIT-F), 4 sessions	1	86	-2.20	-5.49, 1.09	0.19	NA	NA	NA
9 M.D. Anderson Symptom Inventory (MDASI), 6 sessions	1	58	-11.17	-29.30, 6.96	0.23	NA	NA	NA
10 Pennebaker’s Inventory of Limbic Languidness (PILL), 6 sessions	1	58	-6.76	-21.35, 7.83	0.36	NA	NA	NA
11 Physical health measure, 1 session	1	80	-0.26	-0.51, -0.01	0.04	NA	NA	NA

NA, not applicable.

As for secondary outcomes in the 22643-month follow-up group, medical appointments for cancer-related morbidities (MD, -1.69, 95% CI, -2.30 to -1.08, P<0.00001), worst pain (MD, 1.76, 95% CI, 0.27 to 3.25, P = 0.02) and physical symptoms (MD, -0.26, 95% CI, -0.51 to -0.01, P = 0.04) showed a significantly beneficial effect on physical health. However, no significant effects were observed in the following outcomes: least pain, average pain, pain interference, sleep quality and fatigue. In the >3-month follow-up group, there was only one study with a 9-month follow-up (Table 3). Analysis of the combined effect of EW on the physical symptoms using a fixed-effect model demonstrated no significant impact of EW on the physical symptoms in BC patients (P>0.05).

**Table 3:** EW intervention and physical health in >3-month follow-up group.

	Number of		Effect size			Heterogeneity		
Outcome	comparisons	participants	MD	95% Cl	P value	χ^2^	P value	I^2^ (%)
11 Physical health measure, 1 session	1	80	-0.02	-0.26, 0.22	0.87	NA	NA	NA

NA, not applicable.

#### Expressive writing intervention and psychological health outcomes

**Fig 5:**
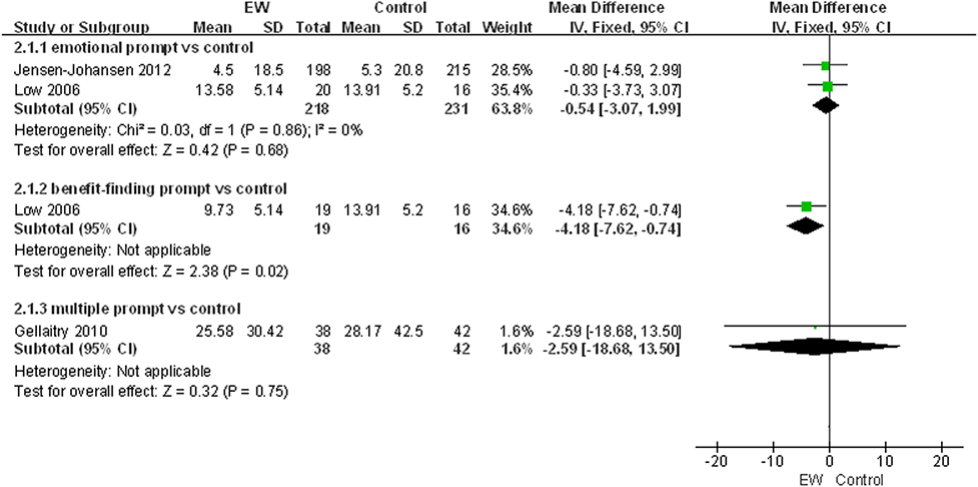
EW intervention and psychological health in ≤3-month follow-up group: negative mood based on POMS.

**Fig 6:**
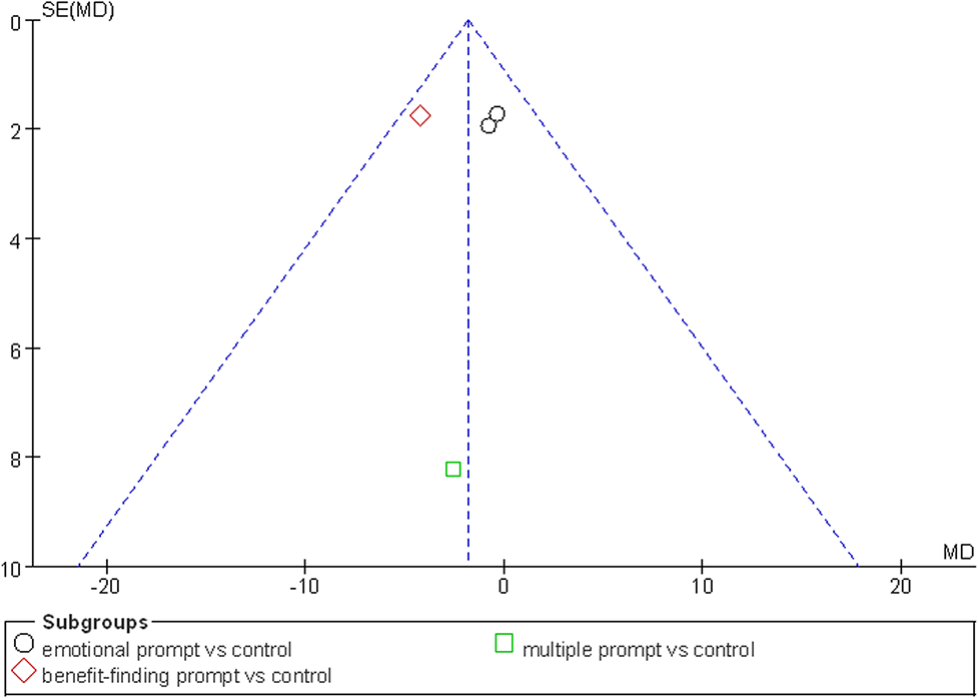
Funnel plot for EW intervention and psychological health in ≤3-month follow-up group: negative mood based on POMS.

The influence of EW on the psychological health of BC patients in all the 11 studies [19–29] was analyzed using a fixed-effect model. Our meta-analysis showed no significant effect of EW on the negative mood, our primary outcome for psychological health, in the ≤3-month follow-up group though one study showed that EW with a benefit-finding prompt significantly reduced the negative mood level in BC patients compared with the control group (P = 0.02; Fig 5). A publication bias analysis of the studies involving these indexes was performed via a funnel plot, and the results followed a symmetric distribution, indicating a low publication bias (Fig 6). Furthermore, Egger’s test also showed a low publication bias (P = 0.975). The secondary outcomes, such as positive mood, stress, depression, intrusive thoughts, avoidance, anxiety and quality of life, in the ≤3-month follow-up group showed insignificant effects on the psychological health (P>0.05) (Table 4). In the >3-month follow-up group of five studies (Table 5), a fixed-effect model was used to analyze the combined effect of EW on the psychological health. Their results showed no significant effect of EW on the psychological health (P>0.05).

**Table 4:** EW intervention and psychological health in ≤3-month follow-up group.

	Number of		Effect size			Heterogeneity		
Outcome	comparisons	participants	MD	95% Cl	P value	χ^2^	P value	I^2^ (%)
1 Negative mood: Profile of Mood States (POMS), 3–4 sessions	4			Subtotals only			Subtotals only	
1.1 emotional prompt vs control	2	449	-0.54	-3.07, 1.99	0.68	0.03	0.86	0
1.2 benefit-finding prompt vs control	1	35	-4.18	-7.62, -0.74	0.02	NA	NA	NA
1.3 multiple prompt vs control	1	80	-2.59	-18.68, 13.50	0.75	NA	NA	NA
2 Positive mood: Profile of Mood States (POMS), 4 sessions	2	71	0.16	-0.95, 1.26	0.78	0.24	0.62	0
3 Passive Positive Mood Scale (PPMS), 3 sessions	1	413	-0.40	-1.75, 0.95	0.56	NA	NA	NA
4 Brief Symptom Inventory 18 (BSI-18), 4 sessions	1	49	0.03	-3.20, 3.26	0.99	NA	NA	NA
5 Perceived Stress Scale (PSS), 4 sessions	1	49	0.84	-2.08, 3.76	0.57	NA	NA	NA
6 Beck Depression Inventory-Short Form (BDI-SF), 3 sessions	1	418	-0.50	-1.32, 0.32	0.23	NA	NA	NA
7 Intrusive thoughts: Impact of Event Scale (IES), 3–4 sessions	4	536	-0.50	-.68, 0.68	0.40	1.18	0.76	0
8 Avoidance: Impact of Event Scale (IES), 3 sessions	3	474	-1.09	-2.59, 0.41	0.15	2.42	0.30	17
9 Meaning/ peace: The Functional Assessment of Chronic Illness Therapy-Spiritual Well-being scale (FACIT-Sp), 4 sessions	1	86	-0.98	-2.63, 0.67	0.24	NA	NA	NA
10Demoralization: The Functional Assessment of Chronic Illness Therapy-Spiritual Well-being scale (FACIT-Sp), 4 sessions	1	86	1.57	-3.05, 6.19	0.51	NA	NA	NA
11 Distress Thermometer (DT), 4 sessions	1	86	0.16	-0.85, 1.17	0.76	NA	NA	NA
12 Anxiety: Hospital Anxiety and Depression Scale (HADS-A), 4 sessions	2	144	-0.59	-1.72, 0.54	0.31	0.12	0.73	0
13 Depression: Hospital Anxiety and Depression Scale (HADS), 6 sessions	1	58	2.48	-1.09, 6.05	0.97	NA	NA	NA
14 Center for Epidemiologic Studies-Depression Scale (CES-D), 1–4 sessions	3	228	-0.23	-0.48, 0.03	0.08	0.04	0.98	0
15 Positive affect: Positive and Negative Affect Scale (PANAS), 1–3 sessions	2	53	2.48	-1.09, 6.05	0.17	0.04	0.85	0
16 Negative affect: Positive and Negative Affect Scale (PANAS), 1–3 sessions	2	53	2.14	-0.65, 4.92	0.13	0.18	0.67	0
17 Cancer-Quality of Life (C-QOL), 6 sessions	1	58	1.31	-4.71, 7.33	0.67	NA	NA	NA
18 Functional Assessment of Cancer Therapy-Breast Cancer Version (FACT-B), 4 sessions	3	229	-0.67	-5.01, 3.66	0.76	0.63	0.73	0

NA, not applicable.

**Table 5:** EW intervention and psychological health in >3-month follow-up group.

	Number of		Effect size			Heterogeneity		
Outcome	comparisons	participants	MD	95% Cl	P value	χ^2^	P value	I^2^ (%)
1 Negative mood: Profile of Mood States (POMS), 3–4 sessions	2	511	-2.19	-5.93, 1.55	0.25	0.03	0.85	0
2 Passive Positive Mood Scale (PPMS), 3 sessions	1	431	0.10	-1.19, 1.39	0.88	NA	NA	NA
3 Intrusive thoughts: Impact of Event Scale (IES), 3–4 sessions	3	483	0.56	-0.78, 1.90	0.41	2.41	0.30	17
4 Avoidance: Impact of Event Scale (IES), 3 sessions	3	483	-0.57	-2.07, 0.94	0.46	0.35	0.84	0
5 Positive affect: Positive and Negative Affect Scale (PANAS)	2	53	2.84	-0.64, 6.31	0.11	0.58	0.45	0
6 Negative affect: Positive and Negative Affect Scale (PANAS),	2	53	3.00	-0.33, 6.33	0.08	0.00	1.00	0
7 Center for Epidemiologic Studies-Depression Scale (CES-D), 1 session	1	80	-0.08	-0.35, 0.19	0.56	NA	NA	NA
8 Beck Depression Inventory-Short Form (BDI-SF), 3 sessions	1	435	-0.30	-.14, 0.54	0.48	NA	NA	NA
9 Functional Assessment of Cancer Therapy-Breast Cancer Version (FACT-B), 4 sessions	3	229	2.26	-1.85, 6.37	0.28	1.58	0.45	0

NA, not applicable.

## Discussion

In this systematic review of 11 RCTs exploring the influence of EW on the health outcomes of BC patients, we tried to determine specific efficacy of EW as a potential therapeutic aid. Based on the physical health indexes, we found that the negative somatic symptoms of BC patients (measured by the *Negative Somatic Symptoms Scale*) were significantly relieved after EW intervention for ≤3 months using either an emotional prompt or a benefit-finding prompt compared with the control group This study also identified a significant effect of EW on medical appointments for cancer-related morbidities, worst pain (measured by *Brief Pain Inventory*) and physical symptoms (measured by instrument *Physical Health Measure*) (Table 2). Although negative somatic symptoms, medical appointments for cancer-related morbidities, worst pain and physical symptoms are different indexes adopted by different assessment instruments, they have, in fact, much in common. They all assess status of physical health in BC patients from different points of view or with different focuses or using different terms. Taken together, the RCTs that evaluated these indexes supported the efficacy of EW on relieving negative physical symptoms in BC patients. Consequently, we can safely conclude that EW intervention may benefit the physical health of BC patients. However, we found that this benefit became insignificant >3 months after EW intervention, which means the positive effect of current EW intervention may not last long. This is in agreement with one of Mogk’s conclusions about health effects of EW [54]. However, we believe that the duration of effectiveness of EW intervention is an important issue that needs further investigation. It is associated with intervention methods or dosages or an inborn limitation of EW itself? As for the psychological health for BC patients, we found no significant effectiveness of EW intervention in either >3-month follow-up group or ≤3-month follow-up group except that only one study reported that EW with a benefit-finding prompt for ≤3 months significantly reduced the negative mood level in BC patients. This finding was rather surprising, because as a means of psychological adjustment EW was expected to exert a positive effect on psychological health.

There have been only four meta-analyses available currently on the EW intervention. The above findings are basically consistent with the meta-analysis by Frisina [52] who determined that written emotional disclosure had a significant effect on the physical but not the psychological health of various clinical populations, and with that by Frattaroli [50] who found EW was effective in both healthy and unhealthy people. However, Harris [53] found EW was effective in healthy people but not in samples defined by medical diagnosis or psychological criteria. Our findings are inconsistent with the meta-analysis by Mogk [54] who concluded that EW had minor or no effects on the healthy or unhealthy subjects in their study. Obviously, the diversity of the populations included and the heterogeneity of the meta-analyses are likely a major reason for the inconsistency in the conclusions on the EW intervention. This was also the reason why we decided to conduct a meta-analysis on EW intervention specifically in BC population.

The poor homogeneity of the limited number of current meta-analyses on EW intervention, including ours, reflects a fact that the researches on EW as an adjuvant therapy for patients are still diversified. The assessment instruments, indexes, outcome measures, samples included, indications, intervention methods, and conclusions are all various. Notably, EW has been tested or used chiefly in Caucasians rather than in oriental races. There is a long way to go before EW can be used as a well-developed intervention in clinic.

The poor homogeneity of the RCTs investigating EW intervention also led to the chief limitations of our meta-analysis. Firstly, because of inconsistent outcome measures by different instruments and incomplete data, the majority of the indexes were unable to be pooled for meta-analysis, leading to inclusion of N = 1 tests in the results. For example, of the 11 studies included in this meta-analysis, only five addressed negative mood as an index of psychological health using the same instrument POMS with emotional prompt, benefit-finding prompt or multiple prompts. Of the five studies, only three provided detailed data we were able to retrieve for sub-group analysis of whether different prompts in EW intervention might have an effect on the intervention outcomes (Fig 5). Other studies measured the effects of EW on psychological health using other instruments or other indexes (Table 4). In addition, the limited number of studies available made it very difficult for us to investigate the potential publication bias influencing the analysis. The power of Egger’s test is too low to distinguish chance from real asymmetry. Secondly, inconsistent factors or indexes, such as characteristics of the subjects and writing prompt and cycle of EW intervention, might have caused biases in results. Thirdly, it was infeasible for us to determine more specific efficacy of EW by further sub-group analyses according to age, education or BC staging because the data were lacking or because the sample size was very limited. Moreover, we did not perform manual retrievals due to time and condition limitations. In addition, three studies failed to provide complete data for five observation indexes even after consultation with the authors [24,25,28], and the full text and data of three conference papers were unavailable even after we tried to contact their authors. All these might have missed some important data on EW as an intervention in BC patients, leading to possible biases in our results.

In summary, this meta-analysis has found that EW intervention may have a positive effect on the physical health rather than the psychological health of BC patients. It proves to chiefly relieve negative physical symptoms in a short term rather than in a long term. The non-lasting momentum of EW intervention is an interesting issue to explore. As a convenient adjunctive intervention for BC patients, EW has much more to be clarified. To determine if personality, race, age, education, BC stage, intervention time since diagnosis and other possible factors would be associated with the intervention effect of EW, it is necessary to do more research in large populations of BC patients to develop EW as an established intervention.

## Supporting Information

S1 Checklist PRISMA 2009 checklist in this meta-analysis.doc. (DOC)

S1 Appendix Search strategy.doc. (DOC)
