# Supplementary material for: Effect of Expressive Writing Intervention on Health Outcomes in Breast Cancer Patients: A Systematic Review and Meta-Analysis of Randomized Controlled Trials
Source: PLoS One. 2015 Jul 7;10(7):e0131802. doi: 10.1371/journal.pone.0131802 (PMC4494859; doi:10.1371/journal.pone.0131802)
Supplement: S1 Appendix — (DOC) [file pone.0131802.s002.doc]

**Search strategy for PubMed (1986-June 2014)**

#1. breast neoplasms

#2. breast malignan* OR breast neoplasm* OR breast cancer* OR breast carcinoma* OR breast adenocarcinoma OR breast tumor* OR breast sarcoma* OR breast lymphedema OR breast dcis

#3. breast ductal OR breast infiltrating OR breast intraductal OR breast lobular OR breast medullary

#4. #1 OR #2 OR #3

#5. mammary neoplasms

#6. mammary malignan* OR mammary neoplasm* OR mammary cancer* OR mammary carcinoma* OR mammary adenocarcinoma OR mammary tumor* OR mammary sarcoma* OR mammary lymphedema or mammary dcis

#7. mammary ductal OR mammary infiltrating OR mammary intraductal OR mammary lobular OR mammary medullary

#8. #5 OR #6 OR #7

#9. #4 OR #8

#10. writing OR written OR expressive OR emotional OR disclosure

#11. #9 AND #10

#12. clinical trial OR trial

#13. random OR randomly OR randomized

#14. #12 OR #13

#15. #11 AND #14

**Search strategy for Web of Science (1986-June 2014)**

#1. TS=breast neoplasms

#2. TS=(breast malignan* OR breast neoplasm* OR breast cancer* OR breast carcinoma* OR breast adenocarcinoma OR breast tumor* OR breast sarcoma* OR breast lymphedema OR breast dcis)

#3. TS=(breast ductal OR breast infiltrating OR breast intraductal OR breast lobular OR breast medullary)

#4. #1 OR #2 OR #3

#5. TS=(mammary neoplasms)

#6. TS=(mammary malignan* OR mammary neoplasm* OR mammary cancer* OR mammary carcinoma* OR mammary adenocarcinoma OR mammary tumor* OR mammary sarcoma* OR mammary lymphedema or mammary dcis)

#7. TS=(mammary ductal OR mammary infiltrating OR mammary intraductal OR mammary lobular OR mammary medullary)

#8. #5 OR #6 OR #7

#9. #4 OR #8

#10. TS=(writing OR written OR expressive OR emotional OR disclosure)

#11. #9 AND #10

#12. TS=(clinical trial OR trial)

#13. TS=(random OR randomly OR randomized)

#14. #12 OR #13

#15. #11 AND #14

**Search strategy for The Cochrane Library (1986-June 2014)**

#1. breast neoplasms

#2. breast malignan* OR breast neoplasm* OR breast cancer* OR breast carcinoma* OR breast adenocarcinoma OR breast tumor* OR breast sarcoma* OR breast lymphedema OR breast dcis

#3. breast ductal OR breast infiltrating OR breast intraductal OR breast lobular OR breast medullary

#4. #1 OR #2 OR #3

#5. mammary neoplasms

#6. mammary malignan* OR mammary neoplasm* OR mammary cancer* OR mammary carcinoma* OR mammary adenocarcinoma OR mammary tumor* OR mammary sarcoma* OR mammary lymphedema or mammary dcis

#7. mammary ductal OR mammary infiltrating OR mammary intraductal OR mammary lobular OR mammary medullary

#8. #5 OR #6 OR #7

#9. #4 OR #8

#10. writing OR written OR expressive OR emotional OR disclosure

#11. #9 AND #10

#12. clinical trial OR trial

#13. random OR randomly OR randomized

#14. #12 OR #13

#15. #11 AND #14

**Search strategy for EMBASE (1986-June 2014)**

#1. 'breast'/exp OR breast AND ('neoplasms'/exp OR neoplasms)

#2. 'breast'/exp OR breast AND malignan* OR 'breast'/exp OR breast AND neoplasm* OR 'breast'/exp OR breast AND cancer* OR 'breast'/exp OR breast AND carcinoma* OR 'breast'/exp OR breast AND ('adenocarcinoma'/exp OR adenocarcinoma) OR 'breast'/exp OR breast AND tumor* OR 'breast'/exp OR breast AND sarcoma* OR 'breast'/exp OR breast AND ('lymphedema'/exp OR lymphedema) OR 'breast'/exp OR breast AND ('dcis'/exp OR dcis)

#3. 'breast'/exp OR breast AND ductal OR 'breast'/exp OR breast AND infiltrating OR 'breast'/exp OR breast AND intraductal OR 'breast'/exp OR breast AND lobular OR 'breast'/exp OR breast AND medullary

#4. #1 OR #2 OR #3

#5. mammary AND ('neoplasms'/exp OR neoplasms)

#6. mammary AND malignan* OR mammary AND neoplasm* OR mammary AND cancer* OR mammary AND carcinoma* OR mammary AND ('adenocarcinoma'/exp OR adenocarcinoma) OR mammary AND tumor* OR mammary AND sarcoma* OR mammary AND ('lymphedema'/exp OR lymphedema) OR mammary AND ('dcis'/exp OR dcis)

#7. mammary AND ductal OR mammary AND infiltrating OR mammary AND intraductal OR mammary AND lobular OR mammary AND medullary

#8. #5 OR #6 OR #7

#9. #4 OR #8

#10. 'writing'/exp OR writing OR written OR expressive OR emotional OR 'disclosure'/exp OR disclosure

#11. #9 AND #10

#12. clinical AND trial OR trial

#13. random OR randomly OR randomized

#14. #12 OR #13

#15. #11 AND #14

**Search strategy for CINAHL (1986-June 2014)**

S1. breast neoplasms

S2. breast malignan* OR breast neoplasm* OR breast cancer* OR breast carcinoma* OR breast adenocarcinoma OR breast tumor* OR breast sarcoma* OR breast lymphedema OR breast dcis

S3. breast ductal OR breast infiltrating OR breast intraductal OR breast lobular OR breast medullary

S4. S1 OR S2 OR S3

S5. mammary neoplasms

S6. mammary malignan* OR mammary neoplasm* OR mammary cancer* OR mammary carcinoma* OR mammary adenocarcinoma OR mammary tumor* OR mammary sarcoma* OR mammary lymphedema or mammary dcis

S7. mammary ductal OR mammary infiltrating OR mammary intraductal OR mammary lobular OR mammary medullary

S8. S5 OR S6 OR S7

S9. S4 OR S8

S10. writing OR written OR expressive OR emotional OR disclosure

S11. S9 AND S10

S12. clinical trial OR trial

S13. random OR randomly OR randomized

S14. S12 OR S13

S15. S11 AND S14
